# Supplementary figures and images for: Potential transoceanic dispersal of Geodia cf. papyracea and six new tetractinellid sponge species descriptions within the Hawaiian reef cryptofauna
Source: PeerJ. 2025 Feb 17;13:e18903. doi: 10.7717/peerj.18903 (PMC11841599; doi:10.7717/peerj.18903)

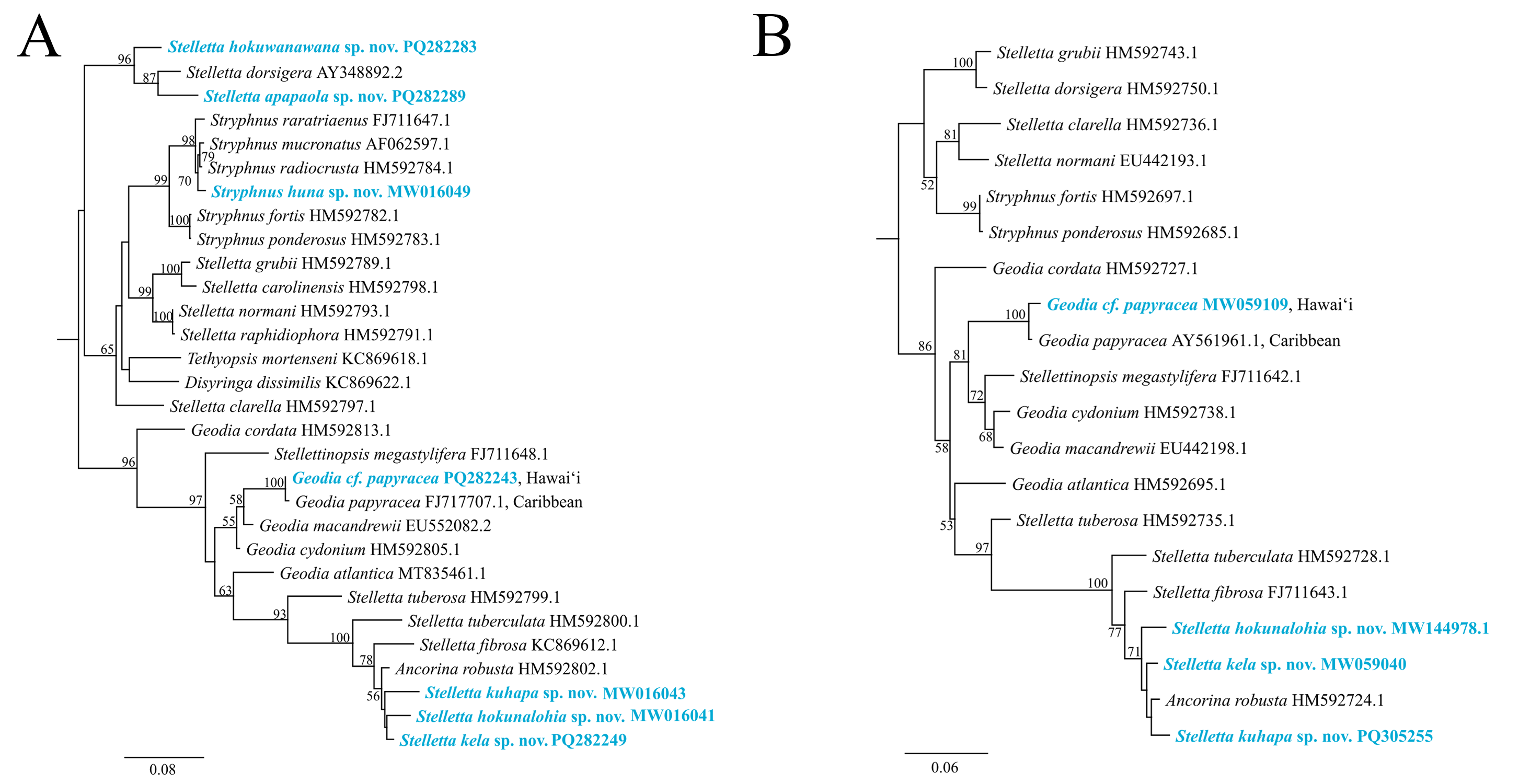

Supplement: Supplemental Information 1 — A, 28S partial sequences from 31 species; B, COI partial sequences from 21 species. This includes sequences generated in this study (blue) and sequences downloaded from GenBank. All GenBank accession numbers are included next to the species names. The 28S and COI alignments consisted of 676 bp and 568 bp respectively. Bootstrap support values >50 are presented at the nodes (2,000 replicates). COI was only successfully obtained for Stelletta kela sp. nov., Stelletta hokunalohia sp. nov., Stelletta kuhapa sp. nov., and Geodia cf. papyracea. Outgroup used,Cinachyrella apion(28S: HM592753.1, COI: HM592667.1). [file peerj-13-18903-s001.png]
