# Supplementary material for: Potential transoceanic dispersal of Geodia cf. papyracea and six new tetractinellid sponge species descriptions within the Hawaiian reef cryptofauna
Source: PeerJ. 2025 Feb 17;13:e18903. doi: 10.7717/peerj.18903 (PMC11841599; doi:10.7717/peerj.18903)
Supplement: Supplemental Information 2 — FMNH refers to the Florida Museum of Natural History and BPBM to the Bernice Pauahi Bishop Museum. * indicates holotype. – indicates no sequence was obtained. [file peerj-13-18903-s002.docx]

| **Species** | **Type** | **FMNH** | **BPBM cat no** | **Field code** | **COI Genbank acc no.** | **28S Genbank acc no.** |  |
| --- | --- | --- | --- | --- | --- | --- | --- |
| *Geodia cf. papyracea* | paratype |  | BPBM C1626 | BKON-2780 | MW059109 | PQ282243 |  |
| *Geodia cf. papyracea* | paratype |  | BPBM C1736 | KB102623_7 | - | PQ282244 |  |
| *Geodia cf. papyracea* | paratype |  | BPBM C1737 | KB102623_8 | - | PQ282245 |  |
| *Stelletta* *apapaola* sp. nov.* | holotype |  | BPBM C1632 | KB042023_1 | - | PQ282288 |  |
| *Stelletta* *apapaola* sp. nov. | paratype |  | BPBM C1631 | 05302023_5 | - | PQ282289 |  |
| *Stelletta* *apapaola* sp. nov. | paratype |  | BPBM C1693 | SLC_080422_54 | - | PQ282290 |  |
| *Stelletta* *apapaola* sp. nov. | paratype |  | BPBM C1694 | SLC_080422_59 | - | PQ282291/PQ282292 |  |
| *Stelletta* *apapaola* sp. nov. | paratype |  | BPBM C1692 | INC_103 | - | PQ282293/PQ282294 |  |
| *Stelletta* *hokunalohia* sp. nov.*** | holotype |  | BPBM C1599 | BKON-2529 | - | PQ282264 |  |
| *Stelletta* *hokunalohia* sp. nov. | paratype |  | BPBM C1604 | OA060717_17 | - | MW016039 |  |
| *Stelletta* *hokunalohia* sp. nov. | paratype |  | BPBM C1606 | OA041017_14 | - | PQ282250 |  |
| *Stelletta* *hokunalohia* sp. nov. | paratype | UF 3973 | BPBM C1601 | OA060717_46/BKON-2572 | - | MW016040 |  |
| *Stelletta* *hokunalohia* sp. nov. | paratype |  | BPBM C1607 | OA080117_36 | - | PQ282251 |  |
| *Stelletta* *hokunalohia* sp. nov. | paratype | UF 3972 | BPBM C1608 | OA092717_64 /BKON-2571 | - | PQ282252 |  |
| *Stelletta* *hokunalohia* sp. nov. | paratype |  | BPBM C1603 | OA092717_66 | PQ305254 | PQ282253 |  |
| *Stelletta* *hokunalohia* sp. nov. | paratype |  | BPBM C1609 | OA021317_41 | - | PQ282254 |  |
| *Stelletta* *hokunalohia* sp. nov. | paratype |  | BPBM C1610 | OA021317_48 | - | PQ282255 |  |
| *Stelletta* *hokunalohia* sp. nov. | paratype |  | BPBM C1616 | OA_END_091 | - | PQ282256 |  |
| *Stelletta* *hokunalohia* sp. nov. | paratype |  | BPBM C1619 | OA_END_115 | - | PQ282257 |  |
| *Stelletta* *hokunalohia* sp. nov. | paratype |  | BPBM C1620 | OA_END_119 | - | PQ282258 |  |
| *Stelletta* *hokunalohia* sp. nov. | paratype |  | BPBM C1617 | OA_END_189 | - | PQ282259 |  |
| *Stelletta* *hokunalohia* sp. nov. | paratype |  | BPBM C1618 | OA_END_221 | - | PQ282260 |  |
| *Stelletta* *hokunalohia* sp. nov. | paratype |  | BPBM C1615 | OA_END_462 | - | PQ282261 |  |
| *Stelletta* *hokunalohia* sp. nov. | paratype |  | BPBM C1621 | OA_END_471 | - | PQ282262 |  |
| *Stelletta* *hokunalohia* sp. nov. | paratype |  | BPBM C1611 | OA031618_38 | - | PQ282263 |  |
| *Stelletta* *hokunalohia* sp. nov. | paratype |  | BPBM C1612 | OA121916_13 | MW144978.1 | - |  |
| *Stelletta* *hokunalohia* sp. nov. | paratype |  | BPBM C1613 | OA121916_16 | MW144971.1 | - |  |
| *Stelletta* *hokunalohia* sp. nov. | paratype | UF 3840 | BPBM C1600 | BKON-1850 | - | MW016038 |  |
| *Stelletta* *hokunalohia* sp. nov. | paratype |  | BPBM C1605 | OA031618_53 | - | PQ282265 |  |
| *Stelletta* *hokunalohia* sp. nov. | paratype |  | BPBM C1735 | OA112117_45 | - | - |  |
| *Stelletta* *hokunalohia* sp. nov. | paratype |  | BPBM C1602 | OA060717_18 | - | MW016041 |  |
| *Stelletta* *hokunalohia* sp. nov. | paratype | UF 3974 | BPBM C1614 | OA092717_29/BKON-2573 | - | - |  |
| *Stelletta* *hokunalohia* sp. nov. | paratype |  | BPBM C1651 | SLC_080422_28 | - | PQ282266 |  |
| *Stelletta* *hokunalohia* sp. nov. | paratype |  | BPBM C1652 | SLC_080422_51 | - | PQ282267 |  |
| *Stelletta* *hokunalohia* sp. nov. | paratype |  | BPBM C1653 | POO_092622_10 | - | PQ282268 |  |
| *Stelletta* *hokunalohia* sp. nov. | paratype |  | BPBM C1654 | POO_32 | - | PQ282269 |  |
| *Stelletta* *hokunalohia* sp. nov. | paratype |  | BPBM C1655 | POO_44 | - | PQ282270 |  |
| *Stelletta* *hokunalohia* sp. nov. | paratype |  | BPBM C1656 | POO_50 | - | PQ282271 |  |
| *Stelletta* *hokunalohia* sp. nov. | paratype |  | BPBM C1657 | INC_1 | - | PQ282272 |  |
| *Stelletta* *hokunalohia* sp. nov. | paratype |  | BPBM C1658 | INC_47 | - | PQ282273 |  |
| *Stelletta* *hokunalohia* sp. nov. | paratype |  | BPBM C1659 | INC_66 | - | PQ282274 |  |
| *Stelletta* *hokunalohia* sp. nov. | paratype |  | BPBM C1660 | INC_79 | - | PQ282275 |  |
| *Stelletta* *hokuwanawana* sp. nov.* | holotype |  | BPBM C1634 | BKON-2889 | - | PQ282283 |  |
| *Stelletta* *hokuwanawana* sp. nov. | paratype |  | BPBM C1630 | 05302023_4 | - | PQ282284 |  |
| *Stelletta* *hokuwanawana* sp. nov. | paratype |  | BPBM C1691 | POO_35 | - | PQ282285/PQ282286 |  |
| *Stelletta* *hokuwanawana* sp. nov. | paratype |  | BPBM C1633 | 05302023_1 | - | PQ282287 |  |
| *Stelletta* *kela* sp. nov.* | holotype | UF 3970 | BPBM C1644 | OA060717_30/BKON-2569 | - | MW016372 |  |
| *Stelletta* *kela* sp. nov. | paratype |  | BPBM C1643 | OA041017_4 | - | MW016374 |  |
| *Stelletta* *kela* sp. nov. | paratype |  | BPBM C1645 | OA031618_47 | - | - |  |
| *Stelletta* *kela* sp. nov. | paratype |  | BPBM C1635 | OA_END_090 | - | MW016373 |  |
| *Stelletta* *kela* sp. nov. | paratype |  | BPBM C1646 | OA_END_099 | - | PQ282246 |  |
| *Stelletta* *kela* sp. nov. | paratype |  | BPBM C1647 | OA031618_50 | - | PQ282247 |  |
| *Stelletta* *kela* sp. nov. | paratype |  | BPBM C1648 | OA031618_68 | - | PQ282248 |  |
| *Stelletta* *kela* sp. nov. | paratype |  | BPBM C1649 | OA121916_29 | MW059040 | PQ282249 |  |
| *Stelletta* *kela* sp. nov. | paratype | UF 3971 | BPBM C1650 | OA092717_55/BKON-2570 | PQ305253 | MW016037/PQ282295 |  |
| *Stelletta* *kuhapa* sp. nov.*** | | holotype | UF 3978 | BPBM C1666 | OA092717_65/BKON-2577 | PQ305258 | MW016047 |
| *Stelletta* *kuhapa* sp. nov. | | paratype | UF 3975 | BPBM C1661 | OA080117_50/BKON-2574 | PQ305255 | MW016043 |
| *Stelletta* *kuhapa* sp. nov.. | | paratype |  | BPBM C1662 | OA021317_35 | - | MW016042 |
| *Stelletta* *kuhapa* sp. nov. | | paratype |  | BPBM C1663 | OA092717_24 | PQ305256 | MW016044 |
| *Stelletta* *kuhapa* sp. nov. | | paratype | UF 3977 | BPBM C1664 | OA092717_73/BKON-2576 | - | MW016048 |
| *Stelletta* *kuhapa* sp. nov. | | paratype | UF 3976 | BPBM C1665 | OA092717_32/BKON-2575 | PQ305257 | MW016046 |
| *Stelletta* *kuhapa* sp. nov. | | paratype |  | BPBM C1667 | OA080117_42 | - | PQ282276 |
| *Stelletta* *kuhapa* sp. nov. | | paratype |  | BPBM C1670 | OA031618_56 | - | PQ282277 |
| *Stelletta* *kuhapa* sp. nov. | | paratype |  | BPBM C1671 | OA031618_75 | - | PQ282278/PQ282279 |
| *Stelletta* *kuhapa* sp. nov. | | paratype |  | BPBM C1687 | OA_END_190 | - | PQ282280 |
| *Stelletta* *kuhapa* sp. nov. | | paratype |  | BPBM C1688 | OA_END_188 | - | MW016045 |
| *Stelletta* *kuhapa* sp. nov.. | | paratype |  | BPBM C1684 | OA031618_77 | - | PQ282281 |
| *Stelletta* *kuhapa* sp. nov. | | paratype |  | BPBM C1685 | OA031618_67 | - | PQ282282 |
| *Stryphnus huna* sp. nov.* | holotype |  | BPBM C1690 | OA_END_486 | - | MW016049 |  |
