# Supplementary material for: Potential transoceanic dispersal of Geodia cf. papyracea and six new tetractinellid sponge species descriptions within the Hawaiian reef cryptofauna
Source: PeerJ. 2025 Feb 17;13:e18903. doi: 10.7717/peerj.18903 (PMC11841599; doi:10.7717/peerj.18903)
Supplement: Supplemental Information 3 [file peerj-13-18903-s003.docx]

| **Species** | **External morphology** | **Megascleres** | **Microscleres** | **Site description** | **Distribution** |
| --- | --- | --- | --- | --- | --- |
| *Asteropus kaena* (de Laubenfels, 1957) | 2 specimens, 1. drab in color, lighter interior; 2. black, consistency cartilaginous, rough surface | **Plagiotriaenes:** none  **Orthotriaenes:** none  **Anatriaenes:** none  **Oxeas:** 1. 2000–2400 x 36–42; 2. 1000 x 14 μm | **Oxyeuasters:** 16–20 μm in diam.  **Streptasters:** 12 μm | 200 m, dredged | Eastern Indo-Pacific: Oʻahu, Hawaiʻi |
| *Erylus caliculatus* (Lendenfeld, 1910) | Irregular, oval, off-white in color. | **Plagiotriaenes:** none **Orthotriaenes:** rhabdome 200-300 x 13-20 μm; cladome 125-220 μm **Anatriaenes:** none **Oxeas/Styles:** 410-850 x 10-19 μm | **Microrhabds:** 39-52 x 3-5 μm  **Acanthotylasters:** 4-8 rays, 17-50 μm in diam.  **Oxyasters:** 10-20 rays, 9-18 μm in diam.  **Aspidasters:** 72 x 88 x 67-77 μm | 152-206 m, coral, sand, shells | Eastern Indo-Pacific: Hawaiʻi Island, Hawaiʻi |
| *Erylus proximus* (Dendy, 1916) (*sensu*: de Laubenfels, 1951) | Amorphous, color is dark grey to black, endosome is pale gray. Consistency cartilaginous, surface is microtuberculate. large pores, oscules rare | **Plagiotriaenes:** none  **Orthotriaenes:** none  **Anatriaenes:** none  **Oxeas:** 2 sizes, I: 420–500 x 8–12 μm; II: 50 x 3 μm  **Calthrops:** 105–200 x 5–15 μm | **Aspidasters:** less than 10 μm thick and 80 μm in diam. **Eutylasters:** 15 μm in diam. | 50 m, ocean floor/dredged | Eastern Indo-Pacific: Hawaiʻi Island, Hawaiʻi; Western Indo-Pacific: Indian Ocean, Red Sea; Temperate Australasia: Houtman Abrolhos |
| *Erylus rotundus* (Lendenfeld, 1910) | Massive, oval or irregular, lobose, white, light brown, purple-brown/black in color | **Plagiotriaenes:** rhabdome 170-370 x 6-12 μm; cladome 80-270 μm in diam.* **Orthotriaenes:** rhabdome 170-370 x 6-12 μm ; cladome 80-270 μm in diam.* **Anatriaenes:** none **Oxeas:** 310-650 x 6-15 μm | **Microrhabds:** 30-98 x 1.5-7.5 μm  **Acanthotylasters:** 2-14 rays, 12-31 μm in diam.  **Oxyasters:** 8-22 rays, 7-17 μm in diam.  **Aspidasters:** 50 x 77 x 46-70 μm | 44-473 m, sand, shells, corals | Eastern Indo-Pacific: Molokai, Kauai, Hawaiʻi Island, Hawaiʻi |
| *Erylus rotundus* var. *cidaris* (Lendenfeld, 1910) | Massive, oval or irregular, lobose, white, light brown, purple-brown/black in color | **Plagiotriaenes:** rhabdome 180-190 μm; cladome 300-440 μm in diam.* **Orthotriaenes:** rhabdome 180-190 μm; cladome 300-440 μm in diam.* **Anatriaenes:** none **Oxeas:** 440-650 x 8-12 μm | **Microrhabds:** 32-50 x 2-4.5 μm  **Acanthotylasters:** >2 rays, 12-31 μm in diam.  **Oxyasters:** 8-14 μm in diam.  **Aspidasters:** 65-75 x 62-69 μm | 44-473 m, sand, shells, corals | Eastern Indo-Pacific: Molokai, Hawaiʻi |
| *Erylus rotundus* var. *megarhabdus* (Lendenfeld, 1910) | Massive, oval or irregular, lobose, white, light brown, purple-brown/black in color | **Plagiotriaenes:** rhabdome 170-370 μm; cladome 160-350 μm in diam.* **Orthotriaenes:** rhabdome 170-370 μm; cladome 160-350 μm in diam.* **Anatriaenes:** none **Oxeas:** 330-650 x 6-13 μm | **Microrhabds:** 43-98 x 3-7.5 μm  **Acanthotylasters: >**3 rays, 12-27 μm in diam.  **Oxyasters:** 7.5-17 μm in diam.  **Aspidasters:** 55-66 x 46-59 μm | 44-473 m, sand, shells, corals | Eastern Indo-Pacific: Kauai, Molokai, Hawaiʻi |
| *Erylus rotundus* var. *typicus* (Lendenfeld, 1910) | Massive, oval or irregular, lobose, white, light brown, purple-brown/black in color | **Plagiotriaenes:** rhabdome 200-220 μm; cladome 150-400 μm in diam.* **Orthotriaenes:** rhabdome 200-220 μm; cladome 150-400 μm in diam.* **Anatriaenes:** none **Oxeas:** 310-570 x 6-15 μm | **Microrhabds:** 30-66 x 1.5-4 μm  **Acanthotylasters:** >3 rays, 15-31 μm in diam.  **Oxyasters:** 7-16 μm in diam  **Aspidasters:** 50-77 x 48-70 μm | 44-473 m, sand, shells, corals | Eastern Indo-Pacific: Kauai, Molokai, Hawaiʻi Island, Hawaiʻi |
| *Erylus sollasi* (Lendenfeld, 1910) | Irregularly massive, lobose, white to brown or purple brown, oscules 1-3 mm in diam. | **Plagiotriaenes:** rhabdome 140-520 x 8-22 μm; cladome 120-300 μm in diam.*  **Orthotriaenes:** rhabdome 140-520 x 8-22 μm; cladome 120-300 μm in diam.*  **Anatriaenes:** none  **Oxeas/Styles:** 425-980 x 8-24 μm  **Dichotriaenes:** rhabdome 140-520 x 8-22 μm; cladome 70-270 μm in diam. | **Microrhabds:** 30-78 x 2.5-5 μm  **Acanthotylasters:** 2-14 rays, 10-38 μm in diam.  **Aspidaster:** 95-156 x 55-82 μm | 42-207 m, sand shells, stone, coral | Eastern Indo-Pacific: Molokai, Hawaiʻi Island, Hawaiʻi |
| *Geodia gibberella* (de Laubenfels, 1951) | massive, subglobular, color is gray, hard surface, soft interior | **Plagiotriaenes:** rhabdome 410 x 18 μm, cladome 60 μm in diam. **Orthotriaenes:** none **Anatriaenes:** none **Oxeas:** 15 x 620 μm | **Sterrasters:** 37 μm in diam. **Oxyeuasters:** 6 μm in diam. O**xyspherasters:** 5 μm in diam. | 3 m, growing on coral | Eastern Indo-Pacific: Hawaiʻi Island, Hawaiʻi |
| *Jaspis digonoxea* (de Laubenfels, 1950) | Encrusting, yellowish-gray in color, cartilaginous/firm consistency with smooth surface. Dense interior with cavities >40 μm in diam.. Flagellate chambers (25 μm in diam.) | **Plagiotriaenes:** none **Orthotriaenes**: none **Anatriaenes:** none **Oxeas:** 400–520 x 7–12 μm, rare | **Oxyeuaster:** 10–20 μm in diam. **Twice-bent microxea:** 105 x 3 μm | 2–8 m, on dead coral | Eastern Indo-Pacific: Oʻahu, Hawaiʻi; Western Indo-Pacific: South Africa |
| *Jaspis pleopora* (de Laubenfels, 1957) | 2 specimens, 1. thin crust; 2. massive; Both bright yellow live, olive green in ethanol, cartilaginous consistency, rough surface. no oscula visible | **Plagiotriaenes:** none **Orthotriaenes:** none **Anatriaenes:** none **Oxeas:** 600–800 x 6–8 μm | **Spherasters:** with smooth, sharp rays, 7 to 20 μm in diam. **Oxyeuasters:** 10 μm in diam., 6–8 rays. | 1. 50 m, dredged; 2. 200 m dredged | Eastern Indo-Pacific: Oʻahu, Hawaiʻi |
| *Stelletta apapaola* sp. nov. | Thinly encrusting, even surface, firm consistency, oscula rare but visible, subsurface channels span the length of the sponge surface. color varies between white and light grey with a tan choanosome. In ethanol, the sponge is grey or white with a white interior. | **Plagiotriaenes:** none  **Orthotriaenes:** none  **Anatriaenes:** none  **Oxeas:** 424–689–900 x 10–24–42 μm.  **Styles:** rare, 519–641–788 x 17–25–36 μm | **Oxyspherasters:** ~15–20 rays, thick, smooth centrum 10–13–16 μm in diam. Rays smooth with infrequent spikes on the tip **Acanthospherasters:** ~15–20 rays, thick centrum, 10–12–13 μm in diam. Arms short, spiked, with smooth centrum | 0.1–1 m, ARMS in a shallow reef habitat | Eastern Indo-Pacific: Oʻahu, Hawaiʻi |
| *Stelletta debilis* (Thiele, 1900) (*sensu*: de Laubenfels, 1951) | massive, pale, consistency cartilaginous, surface smooth and hispid | **Plagiotriaenes:** rhabdome 320 x 16 μm, cladome 32 μm in diam. **Orthotriaenes:** none **Anatriaenes**: present but all broken **Oxeas:** 18 by 720 μm | **Oxyeuasters:** rare, 10 μm in diam. | 1-6 m, growing on dead coral | Eastern Indo-Pacific: Hawaiʻi Island, Hawaiʻi; Central Indo-Pacific: Halmahera |
| *Stelletta hokunalohia* sp. nov. | globular, mostly irregular-shaped, bumpy surface, firm consistency, oscula visible, color varies between white, tan, and purple, choanosome is cream. In ethanol, sponge is white, beige, or light gray. | **Plagiotriaenes:** rhabdome 293–509–696 x 6–19–31 μm; cladome 30–124–178 μm in diam. **Orthotriaenes:** none  **Anatriaenes:** rhabdome 294–445–636 x 5–9–12 μm; cladome 28–38–53 μm in diam.  **Oxeas:** 421–636–861 x 7–13–18 μm | none | 1–4 m, patch reef, ARMS in a shallow reef, and ARMS in mesocosms. | Eastern Indo-Pacific: Oʻahu, Hawaiʻi |
| *Stelletta hokuwanawana* sp. nov. | Thin to thick, irregularly shaped encrustation, hispid surface, tough consistency, oscula visible, color varies between dark grey, white, and beige, choanosome is tan. In ethanol, the cortex is dark grey and the choanosome is white. | **Plagiotriaenes:** none  **Orthotriaenes:** none  **Anatriaenes:** none  **Oxeas:** 536–845–1090 x 14–32–51 μm;  **Styles:** rare, 817 x 42 μm | **Acanthospherasters:** ~15–20 rays, thick centrum exceeding the length of the arms, arms blunt and spiked, smooth centrum, 6–8–9 μm in diam. | 0.1–1 m, ARMS in a shallow reef | Eastern Indo-Pacific: Oʻahu, Hawaiʻi |
| *Stelletta kela*  sp. nov. | Globular to irregular-shaped. hispid surface, tough consistency, oscula visible, color varies between white, brown, red, and grey, choanosome is cream. In ethanol, the sponge is white or tan. | **Plagiotriaenes:** rhabdome 112–356–695 x 6–16–28 μm; cladome 44–112–197 μm in diam. **Orthotriaenes:** rhabdome 452–816–1286 x 17–28–40 μm; cladome 129–214–288 μm in diam. **Anatriaenes:** rhabdome 364–926–1723 x 7–14–23 μm; cladome 32–91–133 μm in diam.  **Oxeas:** 383–793–1175 x 5–16–26 μm | **Acanthostrongylasters:** rare, ~10 rays, thick arms with spikes on tips, smooth centrum, 6–10–12 μm in diam. | 0.3 m, ARMS inside mesocosms | Eastern Indo-Pacific: Oʻahu, Hawaiʻi |
| *Stelletta kuhapa* sp. nov. | Thickly encrusting to irregularly shaped, hispid surface, tough consistency, oscula visible, color varies between tan, white, grey, and dark burgundy, choanosome is cream. In ethanol, sponge is white or beige. | **Plagiotriaenes:** rhabdome 275–532–740 x 9–23–36 μm; cladome 59–184–318 μm in diam. **Orthotriaenes**: none  **Anatriaenes:** rhabdome 385–524–765 x 6–14–20 μm, cladome 27–46–63 μm in diam.  **Oxeas:** 452–775–988 x 8–22–30 μm | **Tylasters:** abundant, ~6–11 rays, 5–8–11 μm in diam. | 0.3 m, ARMS inside mesocosms | Eastern Indo-Pacific: Oʻahu, Hawaiʻi |
| *Stryphnus huna* sp. nov. | Thinly encrusting, no oscula visible, hispid/rubbery surface, tough consistency, color is a light greyish-brown. | **Plagiotriaenes:** none **Orthotriaenes:** none **Anatriaenes:** none **Oxeas:** 2 sizes, I: 406–749–968 x 8–16–24 μm and II: 1149–1474–1861 x 38–50–60 μm | **Sanidasters:** 9–17 μm **Streptasters/oxyasters:** rare, 3–10 rays, 14–51 μm in diam. | 3 m, ARMS on reef | Eastern Indo-Pacific: Oʻahu, Hawaiʻi |
