# Supplementary material for: Potential transoceanic dispersal of Geodia cf. papyracea and six new tetractinellid sponge species descriptions within the Hawaiian reef cryptofauna
Source: PeerJ. 2025 Feb 17;13:e18903. doi: 10.7717/peerj.18903 (PMC11841599; doi:10.7717/peerj.18903)
Supplement: Supplemental Information 4 — *indicates plagiotriaenes and orthotriaenes were described as “ortho- plagiotriaenes” with no distinct separation. [file peerj-13-18903-s004.docx]

| **Species** | **External morphology** | **Megascleres** | **Microscleres** | **Site description** | **Distribution** |
| --- | --- | --- | --- | --- | --- |
| *Asteropus arenosus* (van Soest & Beglinger, 2008) | irregular, oscula visible, surface rough, brown in color grey interior | **Plagiotriaenes:** none **Orthotriaenes:** none **Anatriaenes:** none **Oxeas:** I: 858–1894–2777 × 36–61–78 μm; II: 298–455–646 × 4–7–12 μm | **Sanidasters:** 15–19–23 μm **Oxyasters:** small centrum, 5–9 spined rays, occasionally smooth, 27–35–41 μm | 12 m, growing on stones | Western Indo-Pacific: Oman, Arabian Sea |
| *Asteropus haeckeli* (Dendy, 1905) | irregular, projections, rough surface | **Plagiotriaenes:** none **Orthotriaenes:** none **Anatriaenes:** none **Oxeas:** <1900 x 65 μm | **Sanidasters:** 16 μm  **Oxyasters:** no centrum, slightly spined rays, 40 μm in diam. | not mentioned | Western Indo-Pacific: South India, Sri Lanka |
| *Asteropus kaena* (de Laubenfels, 1957) | 2 specimens, 1. drab in color, lighter interior; 2. black, consistency cartilaginous, rough and lumpy surface | **Plagiotriaenes:** none **Orthotriaenes:** none **Anatriaenes:** none **Oxeas:** 1. 2000–2400 x 36–42; 2. 1000 x 14 μm. | **Oxyeuasters:** 16–20 μm in diam. S**treptasters:** 12 μm | 200 m, dredged | Eastern Indo-Pacific: Oʻahu, Hawaiʻi |
| *Asteropus ketostea* (de Laubenfels, 1950) | encrusting, black exterior, grey interior, hard consistency, lumpy surface, oscules not visible | **Plagiotriaenes:** none **Orthotriaenes:** none **Anatriaenes:** none **Oxeas:** 400–1000 x 3–25 μm | **Streptasters:** 12–18 μm **Oxyasters:** 30 μm | 1 m, under a rock | Tropical Atlantic: Bermuda, Colombia, Caribbean |
| *Asteropus moolenbeeki* (van Soest & Beglinger, 2008) | encrusting, rough surface, hard consistency, oscules not visible, in ethanol, black | **Plagiotriaenes:** none **Orthotriaenes:** none **Anatriaenes:** none **Oxeas:** I: 486–594–760 × 7–15–19 μm. II: 881–1053–1295 × 23–28–36 μm. | **Sanidasters:** 12–15.2–18 μm **Oxyasters:** 6–10 spined rays, 21–28–36 μm  **Strongylasters:** 8 rays, spined tips, rare, 13–19 μm | depth not mentioned, growing on stones | Western Indo-Pacific: Oman, Arabian Sea |
| *Asteropus plumos* (Sim & Kim, 1994) | massive, soft, compressible, rough surface, brown in color | **Plagiotriaenes:** none **Orthotriaenes:** none **Anatriaenes:** none **Oxeas:** I: 675–1875 x 13–55 μm, II: 80–180 μm | **Sanidasters:** 13–25 μm **Oxyasters:** 25–55 μm | not mentioned | Temperate Northern Pacific: East China Sea, South Korea |
| *Asteropus simplex* (Carter, 1879) | massive, brown in color, firm surface, no visible oscules | **Plagiotriaenes:** none **Orthotriaenes:** none **Anatriaenes:** none **Oxeas:** 1300 x 25 μm | **Oxyaster:** 2–12 spined rays, 21–58 μm  **Sanidasters:** 16 μm | not mentioned | Western Indo-Pacific, Eastern Indo-Pacific, Temperate Northern Pacific, Central Indo-Pacific, Temperate Australaisa |
| *Asteropus syringifer* (van Soest & Stentoft, 1988) | globular, oscules visible, rough surface, cartilaginous consistency, grey in color | **Plagiotriaenes:** none **Orthotriaenes:** none **Anatriaenes:** none **Oxeas:** 600–1900 x 15–45 μm | **Sanidasters:** 12–20 μm **Oxyasters:** 8–12 rays, 40–60 μm | 100 m, shelly substrate | Tropical Atlantic: Caribbean, Barbados |
| *Jaspis grisea* (Lévi, 1959) | Rigid, massive, brownish grey, oscula visible | **Plagiotriaenes:** none **Orthotriaenes:** none **Anatriaenes:** none **Oxeas:** 450–700 x 20–35 μm | **Oxyasters:** 8 μm in diam. | 12 m, substrate not mentioned | Tropical Atlantic: Principe, West Africa |
| *Stelletta agglutinans* (Dendy, 1905) | spherical, surface is uneven and rough, in ethanol, the sponge is grey with white chaonosome | **Plagiotriaenes:** none **Orthotriaenes:** none **Anatriaenes:** none **Oxeas:** 2500 x 73 μm | **Chiasters:** no centrum, smooth, blunt rays, 12 μm in diam. | not mentioned | Western Indo-Pacific: South India, Sri Lanka |
| *Stelletta anasteria* (Esteves & Muricy, 2005) | thickly encrusting, subspherical, pale beige in color, osculues not visible, surface smooth and rough, consistency firm and compressible | **Plagiotriaenes:** Straight rhabdome, 210–360–491 x 2–6–9 μm; cladome sharply pointed, 23–58–95 μm **Orthotriaenes:** none **Anatriaenes:** rhabdome 202–401–509 x 2–4–5 μm; cladome 11–19–25 μm **Oxeas:** 242–449–559 x 2–5–8 μm | none | 2 m, under a boulder | Tropical Atlantic: Eastern Brazil |
| *Stelletta anthastra* (Lehnert & Stone, 2014) | massive, white in color, elastic consistency, no oscules visible | **Plagiotriaenes:** rhabdome 680–910 x 112–115 μm, cladome 480–500 x 90–100 μm* **Orthotriaenes:** rhabdome 680–910 x 112–115 μm, cladome 480–500 x 90–100 μm* **Anatriaenes:** rhabdome 19,000 x 30 μm, cladome 180 x 20 μm **Oxeas:** 9500 x 11,900 x 50– 120 μm | **Oxyspheraster:** 14–20 μm; anthasters, spiny rays, 5– 8 μm | 225 m, dredged, attached to sand | Temperate Northern Pactific: Aleutian Islands |
| *Stelletta apapaola* sp. nov. | Thinly encrusting, even surface, firm consistency, oscula rare but visible, subsurface channels span the length of the sponge surface. color varies between white and light grey with a tan choanosome. In ethanol, the sponge is grey or white with a white interior. | **Plagiotriaenes:** none  **Orthotriaenes:** none  **Anatriaenes:** none  **Oxeas:** 424–689–900 x 10–24–42 μm.  **Styles:** rare, 519–641–788 x 17–25–36 μm | **Oxyspherasters:** ~15–20 rays, thick, smooth centrum 10–13–16 μm in diam. Rays smooth with infrequent spikes on the tip. **Acanthospheraster:** ~15–20 rays, thick centrum, 10–12–13 μm in diam. Arms short, spiked, with smooth centrum. | 0.1–1 m, ARMS in a shallow reef habitat | Eastern Indo-Pacific: Oʻahu, Hawaiʻi |
| *Stelletta beae* (Hadju & Carvalho, 2003) | subspherical, hispid surface, oscules visible, barely compressible, white, light purple, dark grey in color; in ethanol, white, yellowish-white, or light grey | **Plagiotriaenes:** none **Orthotriaenes:** I: cladome 97–199–310 μm, rhabdome 262–495–737 x 10–41 μm. II: cladome 19–56–112 μm, rhabdome 87–194–315 x 2–10 μm **Anatriaenes:** rhabdome 155–585–766 x 5–13 μm, cladome 7–24–30 μm,  **Oxeas:** 252–664–931 x 6–22 μm | **Tylasters:** long rays, slender, with few spines, 7–11–14μm | 1–2 m, under large boulders | Temperate South America: Southeastern Brazil |
| *Stelletta brevidens* (Topsent, 1897) | yellow in color, globular, uneven surface, hispid | **Plagiotriaenes:** none **Orthotriaenes:** rhabdome, 500–700 μm; cladome, 133–270 μm **Anatriaenes:** rhabdome, 825 μm; cladome: 40 μm **Oxeas:** present, no measurements listed | **Chiasters:** without centrμm, 8–12 rays, 8–10 μm in diam. | <10 m, substrate not mentioned | Central Indo-Pacific: Banda Sea |
| *Stelletta carolinensis* (Wells, Wells & Gray, 1960) | irregular, globular, firm consistency, in ethanol, pink in color, surface hispid, oscules visible | **Plagiotriaenes:** none **Orthotriaenes:** none **Anatriaenes:** none **Oxeas:** 380–1500 x 7–20 μm | **Oxyasters:** I: 6–8 blunt rays, 20 μm; II: 5–8 rays, 30–70 μm | no depth mentioned, attached to a rock | Temperate North Atlantic: North Carolina |
| *Stelletta colombiana* (Wintermann-Kilian & Kilian, 1984) | long, skinny, protuberances, surface hispid, oscula visible, live color is white to dark purple, consistency is firm and tough | **Plagiotriaenes:** none **Orthotriaenes:** none **Anatriaenes:** none **Oxeas:** I: 2400–3000 x 80–100 μm; II: 1300–1600 x 40–50 μm; III: 535–676 x 20–30 μm | **Tylasters:** 5–15 μm in diam. | 25–30 m, attached to rocks | Tropical Atlantic: Colombia |
| *Stelletta debilis* (Thiele, 1900) | brown, globular, large oscula (<2 mm) | **Plagiotriaenes:** none **Orthotriaenes:** rhabdome, 1300 x 60 μm, cladome 200 μm **Anatriaenes:** rhabdome 1450 x 20 μm, cladome 50 μm  **Oxeas:** 1300 x 25 μm | **Oxyasters:** 4–10 rays, 13–25 μm in diameter | not mentioned | Eastern Indo-Pacific: Hawaiʻi Island, Hawaiʻi; Central Indo-Pacific: Halmahera |
| *Stelletta dendyi* (Sollas, 1888) | irregular, hollow cylinder, surface hispid, oscula visible, greyish-white in color | **Plagiotriaenes:** none **Orthotriaenes:** none **Anatriaenes:** none **Oxeas:** Somal oxea, fusiform, 2000 x 97 μm; Ectosomal strongyloxea, cylindrical, 1000 x 13 μm | **Somal spheraster:** small centrum, 2–10 rays, 15–100 μm in diam.  **Ectosomal spheraster:** small centrum, 18–20 rays, 12–40 μm in diam. | 256 m, dredged, in mud | Central Indo-Pacific: Banda Sea |
| *Stelletta dorsigera* (Schmidt, 1864) | outer color is black-brown, tissue is yellow | **Plagiotriaenes:** present but no measurements **Orthotriaenes:** none **Anatriaenes:** present but no measurements **Oxeas:** present but no measurements | Present but no measurements | not mentioned | Temperate Northern Atlantic: Adriatic Sea, Aegean Sea, Mediterranean |
| *Stelletta durissima* (Bergquist, 1965) | grayish-white in color in ethanol. texture crisp, surface is uneven, rough, no oscules visible | **Plagiotriaenes:** rhabdome, 500–835–1398 x 19–38–70 μm; cladome 60–487 μm* **Orthotriaenes:** rhabdome, 500–835–1398 x 19–38–70 μm; cladome 60–487 μm* **Anatriaenes:** rhabdome, 787–1134–1375 x 23–32–37 μm; cladome, 125–150 μm **Oxeas:** I: 587–1009–1460 x 16–32–50 μm; II: 200–223–240 x 2–3–4 μm | **Tylasters:** 5–10 rays, 8–10–11 μm | 2–6 m, growing on limestone and sand | Central Indo-Pacific: Palau, Solomon Archipelago |
| *Stelletta fibrosa* (Schmidt, 1870) (*sensu*: Pulitzer-Finali, 1986) | massive, cream-white and purple in color, consistency firm, slightly compressible, surface is rough, four oscules. | **Plagiotriaenes:** rhabdome, 1100–1300 x 13–29 μm, cladome 75–90 μm **Orthotriaenes:** none **Anatriaenes:** rare, rhabdome, 1170–1290 x 12–23 μm, cladome 50–80 μm **Oxeas:** 1170–1500 x 23–38 μm | **Tylasters:** rare, 5–9 rays, 10–15 μm  **Sclerites:** rare, 8.5–12 μm  **Spheroxyasters:** 11.5–17.5 μm | 1 m, substrate not mentioned | Tropical Atlantic: Puerto Rico, Florida, Gulf of Mexico, Caribbean |
| *Stelletta globulariformis* (Wilson, 1902) | globular, one oscula, surface is uneven, reddish-brown in color | **Plagiotriaenes:** rhabdome, 1000 x 24 μm; cladome, 80 μm **Orthotriaenes:** none **Anatriaenes:** not abundant, rhabdome 1450 x 16 μm; cladome, 52 μm **Oxeas:** 1430 x 27 μm | **Strongylasters:** abundant, 6–12 tylote rays, 12 μm in diam. | 36–42 m, growing on coral | Tropical Atlantic: Puerto Rico, Caribbean |
| *Stelletta hokunalohia* sp. nov. | globular, mostly irregular-shaped, bumpy surface, firm consistency, oscula visible, color varies between white, tan, and purple, choanosome is cream. In ethanol, sponge is white, beige, or light gray. | **Plagiotriaenes:** rhabdome 293–509–696 x 6–19–31 μm; cladome 30–124–178 μm in diam.  **Orthotriaenes:** none  **Anatriaenes:** rhabdome 294–445–636 x 5–9–12 μm; cladome 28–38–53 μm in diam.  **Oxeas:** 421–636–861 x 7–13–18 μm | none | 1–4 m, patch reef, ARMS in a shallow reef, and ARMS in mesocosms. | Eastern Indo-Pacific: Oʻahu, Hawaiʻi |
| *Stelletta hokuwanawana* sp. nov. | Thin to thick, irregularly shaped encrustation, hispid surface, tough consistency, oscula visible, color varies between dark grey, white, and beige, choanosome is tan. In ethanol, the cortex is dark grey and the choanosome is white. | **Plagiotriaenes:** none  **Orthotriaenes:** none  **Anatriaenes:** none  **Oxeas:** 536–845–1090 x 14–32–51 μm;  **Styles:** rare, 817 x 42 μm | **Acanthospherasters:** ~15–20 rays, thick centrum exceeding the length of the arms, arms blunt and spiked, smooth centrum, 6–8–9 μm in diam. | 0.1–1 m, ARMS in a shallow reef | Eastern Indo-Pacific: Oʻahu, Hawaiʻi |
| *Stelletta jonesi* (Thomas, 1973) | semicircular, thinly encrusting, pale white in color, consistency is hard and incompressible, oscules not visible | **Plagiotriaenes:** none **Orthotriaenes:** none **Anatriaenes:** none **Oxeas:** 452–1000 x 8–33 μm | **Choanosomal oxyasters:** centrum small, rays long and sharp, rarely spined, 6–12 rays, 50–96 μm in diam.  **Dermal oxyasters:** 6–12 rays, 4–16 μm in diam. | depth not mentioned, growing on coral | Western Indo-Pacific: Seychelles Bank, Northern Madagascar |
| *Stelletta kela* sp. nov. | Globular to irregular-shaped. hispid surface, tough consistency, oscula visible, color varies between white, brown, red, and grey, choanosome is cream. In ethanol, the sponge is white or tan. | **Plagiotriaenes:** rhabdome 112–356–695 x 6–16–28 μm; cladome 44–112–197 μm in diam.  **Orthotriaenes:** rhabdome 452–816–1286 x 17–28–40 μm; cladome 129–214–288 μm in diam.  **Anatriaenes:** rhabdome 364–926–1723 x 7–14–23 μm; cladome 32–91–133 μm in diam.  **Oxeas:** 383–793–1175 x 5–16–26 μm | **Acanthostrongylasters:** rare, ~10 rays, thick arms with spikes on tips, smooth centrum, 6–10–12 μm in diam. | 0.3 m, ARMS inside mesocosms | Eastern Indo-Pacific: Oʻahu, Hawaiʻi |
| *Stelletta kuhapa* sp. nov. | Thickly encrusting to irregularly shaped, hispid surface, tough consistency, oscula visible, color varies between tan, white, grey, and dark burgundy, choanosome is cream. In ethanol, sponge is white or beige. | **Plagiotriaenes:** rhabdome 275–532–740 x 9–23–36 μm; cladome 59–184–318 μm in diam.  **Orthotriaenes**: none  **Anatriaenes:** rhabdome 385–524–765 x 6–14–20 μm, cladome 27–46–63 μm in diam.  **Oxeas:** 452–775–988 x 8–22–30 μm | **Tylasters:** abundant, ~6–11 rays, 5–8–11 μm in diam. | 0.3 m, ARMS inside mesocosms | Eastern Indo-Pacific: Oʻahu, Hawaiʻi |
| *Stelletta moseleyi* (Sollas, 1888) | globular, single oscula, greyish-white in color | **Plagiotriaenes:** none **Orthotriaenes:** rhabdome 1600 x 50 μm, cladome 20 μm **Anatriaenes:** rhabdome 1600 x 20 μm, cladome 83 μm **Oxeas:** 1200–1400 x 13–16 μm | **Ectosomal chiaster:** small centrμm, 8 μm in diam.  **Choanosomal chiaster:** no centrμm, 5–7 rays, 24 μm in diam. | 5–20 m, substrate not mentioned | Central Indo-Pacific: Northern Great Barrier Reef |
| *Stelletta orthotriaena* (Koltun, 1966) (Lehnert & Stone, 2014) | not mentioned | **Plagiotriaenes:** rhabdome 1000 x 20 μm; cladome, 100 x 20 μm **Orthotriaenes:** rhabdome 4000 x 110 μm; cladome 600 x 110 μm  **Anatriaenes:** rhabdome 3000 x 20 μm; cladome 120 x 20 μm **Oxeas:** 2000 x 30 | **Tylaster:** 10–12 μm in diam. | 93 m, substrate not mentioned | Temperate Northern Pacific: Sea of Japan |
| *Stelletta parva* (Row, 1911) | irregular, massive, osculca visible, hard consistency, in ethanol, color is white, smooth surface | **Plagiotriaenes:** none **Orthotriaenes:** rhabdome 1000–1150 x 23 μm, cladome 200 μm **Anatriaenes:** rhabdome 1000–1280 x 9–15 μm, cladome 100 μm **Oxeas:** I: 1000–2600 x 20 μm; II: 250–370 x 8 μm | **Chiaster:** 6–10 rays, 7 μm in diam. | not mentioned | Western Indo-Pacific: Red Sea |
| *Stelletta paucistellata* (Lévi, 1952) | massive, globular, visible oscules, surface soft | **Plagiotriaenes:** rhabdome, 425–680 μm, cladome, 65–135 μm **Orthotriaenes:** none **Anatriaenes:** rhabdome, 275–700 μm, cladome, 25 μm **Oxeas:** fusiform, 650–850 μm | **Tylasters:** 10 μm in diam. | not mentioned | Tropical Atlantic: Sahelian Upwelling; Temperate Northern Atlantic: Canary Islands |
| *Stelletta pudica* (Wiedenmayer, 1977) | subspherical, irregular, yellow-brown, red-brown, purple-brown, off-white in color, in ethanol color is light tan or grey, consistency is firm and tough, surface smooth, oscula visible | **Plagiotriaenes:** none **Orthotriaenes:** none **Anatriaenes:** none **Oxeas:** 760–894–980 x 8–10–15 μm | **Tylasters:** 12–13 μm in diam. | not mentioned | Tropical Atlantic: Bahamas, Caribbean |
| *Stelletta purpurea* (Ridley, 1884) | subspherical, in ethanol, color is purple | **Plagiotriaenes:** rhabdome, 1400-1600 x 45-60 μm* **Orthotriaenes:** rhabdome, 1400-1600 x 45-60 μm* **Anatriaenes:** rhabdome, 2000 x 35 μm, cladome, 100 μm **Oxeas:** 1500-2000 x 37 μm | **Tylasters:** 7-10 slender arms, 20-25 μm in diam. | 7-66 m, bottom sand, mud, coral | Western Indo-Pacific; Temperate Australasia; Central Indo-Pacific; Temperate Northern Pacific |
| *Stelletta siemensi* (Keller, 1891) (senu: Wilson, 1925) | in ethanol black | **Plagiotriaenes:** none **Orthotriaenes:** 2500 x 6 μm **Anatriaenes:** 4300–4400 x 42 μm **Oxeas:** 4500 x 35 μm | **Oxyasters:** 4 μm  **Microsphere:** 5 μm | not mentioned | Western Indo-Pacific: Red Sea; Central Indo-Pacific: Philippines |
| *Stelletta solidissima* (Wilson, 1902) | flattened lobes, oscula visible, firm consistency, dark brown-purple in color | **Plagiotriaenes:** none **Orthotriaenes:** none **Anatriaenes:** none **Oxeas:** 1000 x 28 μm | **Microxea:** 60 x 3 μm  **Chiasters:** 8–10 μm in diam. | 36–42 m, growing on coral | Tropical Atlantic: Puerto Rico, Caribbean |
| *Stelletta stenospiculata* (Uliczka, 1929) | spherical to egg-shaped, oscula visible, subsurface channels | **Plagiotriaenes:** none **Orthotriaenes:** rhabdome 900–1700 x 8–18 μm, cladome 46–217 μm **Anatriaenes:** rhabdome 1700–2500 x 15–29 μm, cladome 60–77 μm **Oxeas:** 1340–1560 x 10–18 μm | **Oxyaster:** 13–18 μm | not mentioned | Tropical Atlantic: Caribbean |
| *Stelletta toxiastra* (Lévi, 1993) | grey, subspherical, rough surface, oscula visible, | **Plagiotriaenes:** rare, rhabdome, 250–1500 x 15–50 μm, cladome, 90–110 x 25–30 μm **Orthotriaenes:** none **Anatriaenes:** rhabdome, 3500–3700 x 50 μm; cladome, 200 μm **Oxeas:** I: 3700 x 50μm; II: 380–480 x 15–20 μm | **Oxyasters:** 90 μm **Spheroxyasters:** 10–12 μm **Chiasters:** 3–5 μm | 360 m, substrate not mentioned | Central Indo-Pacific: New Caledonia |
| *Stelletta tuberculata* (Carter, 1886) (*sensu*: de Laubenfels, 1954) | encrusting, massive, dark grey alive, pale, yellow-drab-dull pink interior, surface is rough and lumpy, oscules not visible | **Plagiotriaenes:** none  **Orthotriaenes:** none **Anatriaenes:** none **Oxeas:** 900–1155 x 13–18 μm | **Euasters:** 4–5 μm in diam. | 3 m, growing on dead coral | Temperate Australasia; Eastern Indo-Pacific: Marshall islands |
| *Stelletta variabilis* (Wilson, 1902) | globular, in ethanol, white or purple-brown, single oscula visible | **Plagiotriaenes:** rhabdome 500 x 4 μm, cladome 20–24 μm. **Orthotriaenes:** rhabdome 1350–1700 x 12–24 μm; cladome 100–250 μm **Anatriaenes:** rhabdome 1530–2210 x 20–24 μm, cladome 44–84 μm **Oxeas:** I: 1020–1600 x 12–16 μm; II: 168–200 x 6–8 μm | **Chiasters:** rare, small or no centrμm, slightly tylote rays, 8–12 rays, 10–16 μm in diam. | 29–42 m | Tropical Atlantic: Eastern Caribbean, Puerto Rico |
| *Stryphnus huna* sp. nov. | Thinly encrusting, no oscula visible, hispid/rubbery surface, tough consistency, color is a light greyish-brown. | **Plagiotriaenes:** none **Orthotriaenes:** none **Anatriaenes:** none **Oxeas:** 2 sizes, I: 406–749–968 x 8–16–24 μm and II: 1149–1474–1861 x 38–50–60 μm | **Sanidasters:** 9–17 μm  **Streptasters/oxyasters:** rare, 3–10 rays, 14–51 μm in diam. | 3 m, ARMS on reef | Eastern Indo-Pacific: Oʻahu, Hawaiʻi |
| *Stryphnus radiocrusta* (Kennedy, 2000) | Massive, subspherical, in ethanol beige-grey cortex and light beige choanosome, no oscules visible, texture is firm, barely compressible, surface is smooth, even, hispid | **Plagiotriaenes:** none **Orthotriaenes:** none **Anatriaenes:** none **Oxeas:** 530–1730 x 5–44 μm | **Sanidasters:** 9–18 μm  **Oxyasters:** 18–33 μm | 3–6 m, substrate not mentioned | Temperate Australasia: Southeast Australia |
