# Supplementary material for: Potential transoceanic dispersal of Geodia cf. papyracea and six new tetractinellid sponge species descriptions within the Hawaiian reef cryptofauna
Source: PeerJ. 2025 Feb 17;13:e18903. doi: 10.7717/peerj.18903 (PMC11841599; doi:10.7717/peerj.18903)
Supplement: Supplemental Information 5 — *indicates outgroup used. [file peerj-13-18903-s005.docx]

| **Species** | **COI GenBank acc No.** | **28S GenBank acc No.** |
| --- | --- | --- |
| *Ancorina robusta* | HM592724.1 | HM592802.1 |
| *Cinachyrella apion** | HM592667.1 | HM592753.1 |
| *Geodia atlantica* | HM592695.1 | MT835461.1 |
| *Geodia cordata* | HM592727.1 | HM592813.1 |
| *Geodia cydonium* | HM592738.1 | HM592805.1 |
| *Geodia macandrewii* | EU442198.1 | EU552082.2 |
| *Geodia papyracea*, Caribbean | AY561961.1 | FJ717707.1 |
| *Geodia cf. papyracea*, Hawaiʻi | MW059109 | PQ282243 |
| *Stelletta kuhapa* sp. nov. | PQ305255 | MW016043 |
| *Stelletta clarella* | HM592736.1 | HM592797.1 |
| *Stelletta hokunalohia* sp. nov. | MW144978.1 | MW016041 |
| *Stelletta dorsigera* | HM592750.1 | AY348892.2 |
| *Stelletta fibrosa* | FJ711643.1 | KC869612.1 |
| *Stelletta grubii* | HM592743.1 | HM592789.1 |
| *Stelletta kela* sp. nov. | MW059040 | PQ282249 |
| *Stelletta normani* | EU442193.1 | HM592793.1 |
| *Stelletta tuberculata* | HM592728.1 | HM592800.1 |
| *Stelletta tuberosa* | HM592735.1 | HM592799.1 |
| *Stellettinopsis megastylifera* | FJ711642.1 | FJ711648.1 |
| *Stryphnus fortis* | HM592697.1 | HM592782.1 |
| *Stryphnus ponderosus* | HM592685.1 | HM592783.1 |
